# A survey on the seroprevalence of toxocariasis and related risk factors in Eosinophilic children of Northwest Iran

**DOI:** 10.4314/ahs.v22i3.66

**Published:** 2022-09

**Authors:** Arash Pourgholaminejad, Habib Razipour, Peyman Heydarian, Keyhan Ashrafi, Zahra Atrkar Roushan, Meysam Sharifdini

**Affiliations:** 1 Department of Immunology, School of Medicine, Guilan University of Medical Sciences, Rasht, Iran; 2 Department of Medical Parasitology and Mycology, School of Medicine, Guilan University of Medical Sciences, Rasht, Iran; 3 Department of Medical Parasitology and Mycology, School of Medicine, Qazvin University of Medical Sciences, Qazvin, Iran; 4 Department of Biostatistics, School of Medicine, Guilan University of Medical Sciences, Rasht, Iran

**Keywords:** Seroprevalence, Toxocariasis, anti-*Toxocara* antibody, Eosinophilia children, Qazvin, Iran

## Abstract

**Background:**

Toxocariasis is a serious zoonotic helminthic disease caused by the nematodes; *Toxocara* species.

**Aim:**

A cross-sectional study was conducted to determine the seroprevalence of toxocariasis and related risk factors in eosinophilic children referred to the pediatrics hospital of Qazvin province northwest Iran during 2019–2020.

**Methods:**

A total of 200 blood samples were collected from eosinophilic children referred to the Qods Pediatrics Hospital. Demographic data, clinical symptoms, and dogs- and soil-contact history were collected. The presence of anti-*Toxocara* IgG antibody was evaluated by *T. canis* IgG ELISA kit.

**Results:**

Anti-*Toxocara* IgG antibodies were detected in 14 (7%) of the total eosinophilic children. The seropositive rate of toxocariasis in hyper-eosinophilic children (>1000/mm3) was 15.1%, while the seropositivity was 4.1% in children with eosinophilia status (500–999/mm3). There was a significant association between the eosinophilia rate and seropositivity (P<0.05). Also, seroprevalence in asymptomatic eosinophilic children was 4.4%, while in children with clinical symptoms it was 17.1%. Accordingly, a statistically significant difference was found between clinical symptoms and *Toxocara* infection (P<0.05).

**Conclusion:**

The prevalence of toxocariasis in eosinophilic children is a serious health problem in the study area. Therefore, serologic evaluation for the diagnosis of *Toxocara* infection is recommended for eosinophilic children.

## Introduction

Toxocariasis is a zoonotic helminthic infection with a considerable impact on public health particularly, in tropical regions caused by the genus *Toxocara* including *T. canis* and *T. cati* which are intestinal nematodes of dogs and cats, respectively^1^. Humans as paratenic hosts get infected through the fecal-oral route by the accidental ingestion of embryonated eggs through contact with dogs, cats, and soil and also consumption of contaminated raw vegetables or undercooked meat from other paratenic hosts^2^. After ingestion of embryonated eggs, the larvae migrate via the circulatory system to various organs such as lungs, liver, skeletal muscles, heart, eyes, and also central nervous system (CNS). Migrating larvae are attacked by the host immune system, resulting in local inflammatory responses associated with eosinophilia, hyper-cytokinemia, and increased production of specific antibodies^3^.

Clinical manifestations of toxocariasis are a spectrum of symptoms depending on the organs affected, including visceral larva migrans (VLM), ocular larva migrans (OLM), neurologic, and covert toxocariasis^4^. However, the majority of infections remain asymptomatic, granulomatous lesions that likely contained *Toxocara* spp. larvae are observed in organs of some cases^5^. Young children playing in areas contaminated with dog feces and soil are the main population at risk for Toxocara infection^6^. A definitive diagnosis of human toxocariasis relies mainly on the detection of anti-*Toxocara* antibodies by serological approaches^7^.

This neglected tropical disease is prevalent among socioeconomically disadvantaged children, and its seroprevalence is highly variable from 2.4 to 92.8% globally^8^. Accordingly, several studies have described the seropositivity of *T. canis* among Iranian people, around 22% 2,9–13. Based on the global prevalence of this ascarid infection, a high range of up to 82% has been reported in puppies and dogs^14^ however some studies reported the prevalence of 1.9 - 60% in dogs and pets in Iran^15^,^16^. Differences in the seroprevalence of *Toxocara* antibodies in humans might be attributed to geographic location, temporal and climate variations, socio-economic status, range of dogs population as well as dietary habits^17^.

Eosinophilia is a central feature of the host immune response to helminthic parasites^18^, and it occurs against migrating larvae of *Toxocara* species^2^. It is recognized based on the elevation of blood eosinophil absolute counts of more than 500 cells/µl^19^. In severe cases, the blood leukocyte count might reach 100000/mm^3^, while 80–90% are eosinophils^20^. Also, it has been reported that 68% of patients with unknown eosinophilia were diagnosed as having specific anti-*Toxocara* antibodies^21^. It is considering that most patients have no clinical signs and symptoms while the measurement of anti-*Toxocara* antibody is crucial, exclusively in patients with unknown eosinophilia. Due to the wide range of etiologies for eosinophilia, proper diagnosis is necessary for the effective treatment of this concern.

Based on a previous study in Qazvin province, the contamination rate of *Toxocara* spp. eggs was 3.15% in soil and 8.42% in the grass of parks^22^. Also, due to the lack of data on the epidemiological and clinical features of human toxocariasis in Qazvin province, we investigated the seroprevalence of *Toxocara* infection in eosinophilic children and its association with related risk factors in the study area.

## Methods

### Study area and population

**Figure 1:**
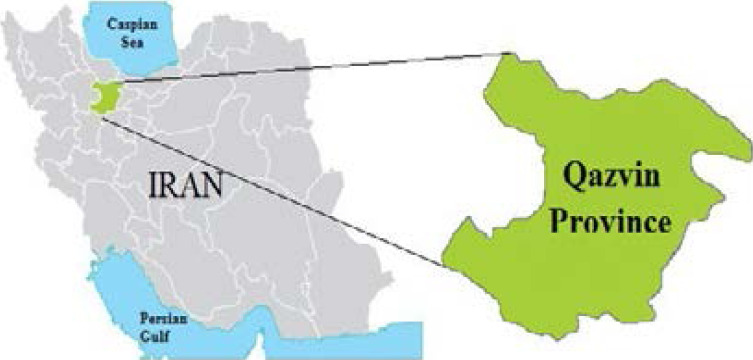
Map of Iran showing the geographical location of Qazvin province.

The area of our study is Qazvin city (36°16′N, 50°00′E), the largest city and capital of the province of Qazvin in Iran. It is located in the northwest of Tehran with a population of around 403000 (Figure 1). The climate of the city is comparatively cold and snowy in winters and temperate in summers with a mean rainfall of 318 mm/year. The temperature typically varies from 24°F to 94°F, and the perceived humidity level in Qazvin does not vary significantly over the course of the year, remaining a virtually constant 0% throughout. Qazvin city is one of the important industrial zones in the country, and most of its population is employed in industrial and institutional jobs while the rural inhabitants usually engaged in farming, gardening, and animal husbandry. Rarely are pet dogs seen in public places in the city, but there are many domestic dogs in the rural areas in close contact with the human population and are used as guards and herding dogs^22^.

This cross-sectional study was carried out on 200 selected eosinophilic children aged 1–13 years, male and female (60.5 and 39.5%) referred to Qods Qazvin Educational and Medical Center for pediatrics. The sampling was carried out randomly (three days per week) between 2019 and 2020. Based on the eosinophilia status, we categorized children into two subgrups; 1) 500–999/µl (eosinophilia) and 2) >1000/µl (hyper-eosinophilia)^23^,^24^.

### Sample size determination

The sample size was determined by statistical formula n = Z^2^×P (1-P)/d^2^ where P (prevalence of Toxocariasis in Iran), d (at 6% marginal error), and standard score (Z) at 95% confidence interval. The prevalence of Toxocariasis in Iran was reported as 22%^2^. The sample size determined for the study was 184.

### Study design

A covering letter explaining the purpose of the study with a structured questionnaire with a consent form for blood sampling was prepared according to the guidelines of the ethics committee of the Guilan University of Medical Sciences. The questionnaire was divided into three sections as follow: 1) demographic information such as age, gender, living location status, parental education, and occupation; 2) disease information such as eosinophilia rate, clinical symptoms (idiopathic fever, ocular, pulmonary, cutaneous, and hepatic disorders); 3) possible risk factors including contact with dogs and soil contact^11^.

### Serologic detection of toxocariasis

Approximately 10 ml peripheral blood was taken from eosinophilic children and used to provide a serum sample. All sera were not hemolytic, lipemic, or icteric and were screened for qualitative determination of *T. canis* specific IgG antibody by using enzyme immunoassay commercial ELISA kit (NovaTec Immundiagnostica GmbH, Germany, Product number: TOCG0450). The diagnostic specificity and sensitivity of the kit are >95%. Briefly, according to the manufacturer's protocol, microtiter strip wells are pre-coated with synthetic *T. canis* antigens to bind corresponding antibodies of the specimen. After washing, horseradish peroxidase (HRP) labeled protein A conjugate was added and the immune complex formed by the bound conjugate was visualized by adding Tetramethylbenzidine (TMB) substrate that gives a blue reaction product. Then, sulphuric acid was added to stop the reaction with a yellow endpoint color. Finally, absorbance at 450/620 nm was measured using an ELISA microwell plate reader.

### Evaluation of results and statistical analysis

To evaluate the results, the NovaTec Units (NTU) was used. We calculated the optical density (OD) of the substrate blank, negative, positive, and cut-off controls. Division of the sample OD by the cut-off value gives the NTU. According to the manufacturer's recommendation, values greater than 11 NTU were considered positive. Chi-square (X2) test was used to compare infection with toxocariasis in association with the related risk factors. Statistical analyses were performed using SPSS (version 23; SPSS Inc, Chicago, USA), and a P-value of ≤ 0.05 was considered statistically significant.

### Ethical approval

The protocol of this study was approved by the Ethics Committee of Guilan University of Medical Sciences, Iran (Ref. No. IR.GUMS.REC.1398.373).

## Results

**Figure 2:**
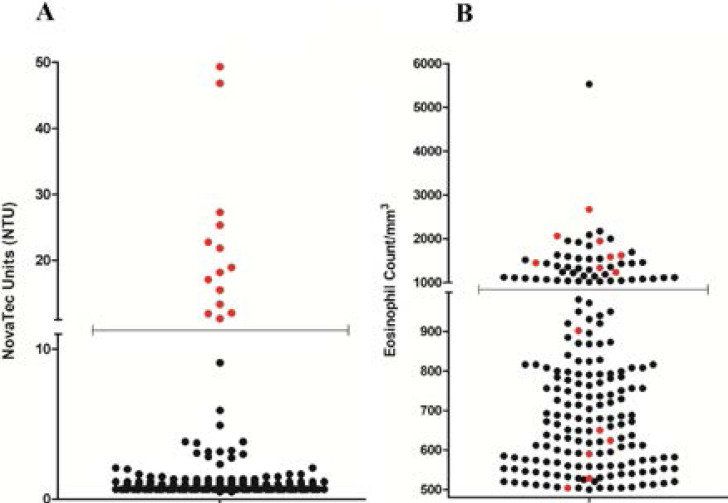
Seroprevalence of *Toxocara canis* within eosinophilic children and the association of *Toxocara* infection with eosinophilia status. A) The scatter plot represents the NTUs of all serum samples obtained from eosinophilic children. Each spot is represented the mean value of independent experiments for every eosinophilic child sample, and each red spot is shown the seropositivity for anti-*Toxocara canis* IgG antibody. The values greater than 11 NTU were considered positive for anti-*Toxocara* IgG antibody according to the manufacturer's datasheet. B) The scatter plot is separated into two segments (500–999/mm3 referred to as eosinophilia and >1000/mm3 referred to as hyper-eosinophilia). It shows the distribution of eosinophilia. Each spot is represented the absolute eosinophil count in the blood sample of every child, and the red spots indicate the status of the seropositive children among other seronegative (black spots) samples. The seropositive rate of toxocariasis in hyper-eosinophilic children was 15.1% (8 out of 53 cases), while the seropositivity was 4.1% (6 out of 147 cases) in children with eosinophilia status.

A total of 200 eosinophilic children were examined in this study, comprising 79 (39.5%) females and 121 (60.5%) males. Anti-*Toxocara* antibodies were detected in sera of 14 (7%) out of 200 subjects (Fig-2A). Furthermore, 8.3% of the males and 5.1% of the females are seropositive, but there was no significant association according to the gender factor (P= 0.386) (Table 1). The participants were categorized into the two groups by age; 1–6 years old (58.5%) and 7–13 years old (41.5%) with 6.03 ± 3.12 mean age. Most of the seropositive cases were in the age group of 1–6 years, and no significant association was found between the age group and *Toxocara* seropositivity (P= 0.404) (Table 1). Although the seropositivity rate in rural areas (9.2%) was higher than in urban areas (5.6%), it also was not significant (P= 0.337).

**Table 1:** The relationship between Toxocara canis seroprevalence and related risk factors and some epidemiological data in eosinophilic children referred to Qods Qazvin Educational and Medical Center. *Salesperson, baker, merchant, grocer, construction worker, mechanic, housekeeper, engineer, taxi driver, chef, tailor, hairstylist, unemployed, etc.

Characteristics	Frequency No. (%)	Sero-Positive No. (%)	Sero-Negative No. (%)	p-value
**Age (years)**				
1–6	117 (58.5)	10 (8.5)	107 (91.5)	**0.404**
7–13	83 (41.5)	4 (4.8)	79 (95.2)	
**Gender**				
Female	79 (39.5)	4 (5.1)	75 (94.9)	**0.386**
Male	121 (60.5)	10 (8.3)	111 (91.7)	
**Parent's Educational** **Level**				
No formal education	5 (2.5)	0 (0)	5 (100)	**1.0**
Primary Education	120 (60)	9 (7.5)	111 (92.5)	
Secondary Education	45 (22.5)	3 (6.7)	42 (93.3)	
Tertiary Education	30 (15)	2 (6.7)	28 (93.3)	
**Parent's Occupation**				
Farmer	10 (5)	0 (0)	10 (100)	**0.572**
Gov. Employer	38 (19)	2 (5.3)	36 (94.7)	
Other*	152 (76)	12 (7.9)	140 (92.1)	
**Living Location**				
Urban	124 (62)	7 (5.6)	117 (94.4)	**0.337**
Rural	76 (38)	7 (9.2)	69 (90.8)	
**Soil Contact**				
Yes	117 (58.5)	7 (6)	110 (94)	**0.579**
No	83 (41.5)	7 (8.4)	76 (91.6)	
**Dog Ownership** **at Home**				
Yes	11 (5.5)	0 (0)	11 (100)	**0.441**
No	189 (94.5)	14 (7.4)	175 (92.6)	
**Blood Eosinophilia** **(cell/mm^3^)**				
500–999	147 (73.5)	6 (4.1)	141 (95.9)	**0.007**
>1000	53 (26.5)	8 (15.1)	45 (84.9)	

Concerning clinical manifestations, the seroprevalence in asymptomatic eosinophilic children was 4.4%, while in children with clinical symptoms, it was 17.1%. There was a significant association between human toxocariasis and clinical symptoms (P= 0.01). Among the eosinophilic children, 7% had experiences idiopathic fever which 21.4% of them were seropositive. Furthermore, in addition to ocular symptoms detected in a seropositive child, 11.5% had pulmonary diseases that 13% of them were seropositive (Table 2).

**Table 2:** The relationship between Toxocara canis seroprevalence and clinical manifestations in eosinophilic children

Clinical Manifestations	Sero-Positive No. (%)	Sero-Negative No. (%)	Total No. (%)	p-value
**Asymptomatic**	7 (50)	152 (81.7)	**159 (79.5)**	**0.01**
**Idiopathic** **Fever**	3 (21.4)	11 (5.9)	**14 (7)**	
**Ocular** **Disease**	1 (7.1)	0 (0)	**1 (0.5)**	
**Pulmonary** **Disease**	3 (21.4)	20 (10.7)	**23 (11.5)**	
**Liver Disease**	0 (0)	1 (0.5)	**1 (0.5)**	
**Cutaneous** **Disease**	0 (0)	2 (1)	**2 (1)**	
**Total**	14 (100)	186 (100)	200 (100)	

The seropositive rate of toxocariasis in hyper-eosinophilic children (>1000/mm^3^) was 15.1% (8 out of 53 cases), while the seropositivity was 4.1% (6 out of 147 cases) in children with eosinophilia status (500–999/mm^3^) (Fig-2B). Accordingly, there was a significant association between seroprevalence of *Toxocara* and blood eosinophilic status (P= 0.007) (Table 1). Furthermore, there was no significant difference in other risk factors including parental occupation and educational status, soil contact, and dog ownership at home (P>0.05).

## Discussion

Toxocariasis is a neglected parasitic zoonosis with a considerable impact on public health especially in disadvantaged communities in some countries^2^. It has variable and non-characteristic manifestations from asymptomatic to complicated forms. Similar to most helminthic infections, toxocariasis causes moderate to severe eosinophilia^5^,^25^. Therefore, hyper-eosinophilia might be the only indication for the diagnosis of toxocariasis in asymptomatic individuals.

In studies on healthy subjects, the prevalence of toxocariasis in different parts of Iran is variable, ranging from 0% to 29.4%^26^,^27^. Also, the prevalence rate in healthy individuals is diverse in different countries. The seroprevalence has been reported with relatively higher exposure levels in some countries including 29.8% in Nigeria^28^, 76.6% in Taiwan^29^, 63.2% in Indonesia^30^, and 50.6% in Brazil^31^. In contrast, the prevalence is lower in developed countries such as 13.9% in USA^32^, 1.6% in Japan^33^, 0.7% in New Zealand^34^, and 2.4% in Denmark^35^.

Several seroepidemiological studies have been performed among eosinophilic individuals in the world, and most of them reported a high seropositive rate for toxocariasis^21^,^36^–^39^. Concerning patients with eosinophilia, the seroprevalence of toxocariasis ranged from 2–23.6% in different areas of Iran^8^. The present study revealed a low positive rate of toxocariasis among eosinophilic subjects compared to most previous reports in the country. According to the present results, there was a statistically significant relationship between the level of blood eosinophilia and the seropositive rate of toxocariasis. In this study, the seroprevalence of *Toxocara* infection in males was higher than in females which is similar to the findings reported from previous studies in, Brazil^31^, Venezuela^40^, Marshall Islands^41^and Croatia^42^. However, it is in contrast with the studies reported from Mexico^43^, Peru^44^, and Brazil^45^. It could be due to the high level of exposure of males to sources of *Toxocara* species infection. Although in our study, the seroprevalence of toxocariasis in preschool children (1–6 years old) was higher than school children (7–13 years old), no significant difference was evident. Our results are similar to those of other studies that showed no significant association between seropositivity and age groups^9^,^40^,^41^. Martínez et al. showed that younger children are considered an at-risk group for toxocariasis because this group has less hygiene and is more likely to play with dogs and soil^46^. It is in contrast to reports from other studies where the highest prevalence was in the oldest groups^43^,^47^.

In the present study, although the majority of seropositive individuals (50%) were asymptomatic, 21.4%, 21.4%, and 7.1% of them had pulmonary symptoms, idiopathic fever, and ocular signs in their clinical manifestation, respectively. It could be related to the asymptomatic nature of toxocariasis^48^. Our results showed a significant relationship between toxocariasis and clinical manifestations. Surprisingly, dog ownership was not associated with the seropositivity of these children. This finding is in agreement with other studies^49^,^50^. Our contradictory result could be due to the low sample size especially, in the children who had dogs at home. However, other researchers indicated a relationship between toxocariasis and the presence of dogs at home^51^,^52^.

In our study, there was no significant association between seroprevalence of *Toxocara* and soil contact. This finding is in agreement with previous studies by Sajjadi et al. 11 and Baghani et al. 53 in Fars and Tehran provinces of Iran but in contrast to those published by other authors that showed a significant association^10^,^51^.

Also, previous studies suggested that the geographic location (urban or rural regions) is a crucial risk factor for the infection due to the high rate of contamination of rural people through soil or dog contact more frequently than those in urban areas^39^,^54^. In this regard, our data showed a positive rate of 9.2% for *T. canis* residing in rural areas, while 5.6% of children living in urban people were seropositive. Although, based on our analyzed results, the locality was not a significant risk factor for toxocariasis. It is probably due to the low sample size in this study.

In this study, the results showed that there was no significant between toxocariasis and the educational level and the occupation of the parents. Similar findings were reported by previous studies^55^,^56^. It might be due to the small number of children and the high diversity of occupations in the study area.

## Conclusion

This is the first study about toxocariasis in school children from the Qazvin province of Iran. The results demonstrated that toxocariasis is currently one of the potential reasons for eosinophilia in children, and physicians should consider it, especially in asymptomatic conditions. Therefore, it should be recommended prevention efforts such as veterinary inspection and anthelmintic treatment of dogs and cats as well as education of personal and food hygiene to children.
